# A Case of Wernicke’s Encephalopathy in a Pregnant Woman With a History of Sleeve Gastrectomy

**DOI:** 10.7759/cureus.9970

**Published:** 2020-08-23

**Authors:** Harika Kandlakunta, Dhineshreddy Gurala, Jobin Philipose, Abhishek Polavarapu, Jeffrey R Abergel

**Affiliations:** 1 Internal Medicine, Northwell Health-Staten Island University Hospital, Staten Island, USA; 2 Gastroenterology and Hepatology, Northwell Health-Staten Island University Hospital, Staten Island, USA

**Keywords:** wernicke's encephalopathy, sleeve gastrectomy, bariatric surgery, thiamine deficiency, pregnant, malabsorption, nutritional deficiency, restrictive surgery

## Abstract

Wernicke’s encephalopathy (WE) is a neurological complication of thiamine deficiency characterized by a triad of acute confusion, ataxia, and ophthalmoplegia. Even though it is most common in chronic alcoholism, an increase in prevalence has been reported recently due to the increased popularity of bariatric surgeries. WE is a known neurological complication after gastric bypass surgery but rarely reported after sleeve gastrectomy. We present a unique case of WE in pregnant women four months after sleeve gastrectomy.

## Introduction

The prevalence of obesity worldwide has doubled since 1980, affecting one-third of the world’s population [1]. Obesity adversely affects physiological functions, quality of life, work productivity, and healthcare costs. Bariatric surgery is recommended for morbid obesity, who have failed conservative measures. Gastric bypass, sleeve gastrectomy, adjustable gastric band, and biliopancreatic diversion with the duodenal switch are the commonly performed weight-loss surgeries in the United States [2]. These procedures can lead to significant complications, including neurological disorders. Wernicke's encephalopathy (WE) is a known neurological complication reported after gastric bypass surgery, due to thiamine deficiency secondary to malabsorption. The incidence of neurological complications after bariatric surgery is 1.18% [3]. Compared to gastric bypass, laparoscopic sleeve gastrectomy is a restrictive procedure that is rarely associated with nutritional deficiencies [4]. We report a unique case of WE due to thiamine deficiency in pregnant women four months after sleeve gastrectomy that improved with thiamine supplementation.

## Case presentation

A 21-year-old female, at twelve weeks of gestation, presented to the emergency department with dizziness and vomiting for one-month. Additionally, she had blurry vision, inattentiveness for the prior four days. The review of systems was otherwise negative. Her past medical history included morbid obesity for which she underwent laparoscopic sleeve gastrectomy four months prior to admission with a successful weight loss of 80 pounds. She was noncompliant with multivitamins after the procedure. There was no history of smoking, alcohol, or other substance abuse. She denied recent use of Tylenol® or herbal supplements. Family history was unremarkable.

**Figure 1:**
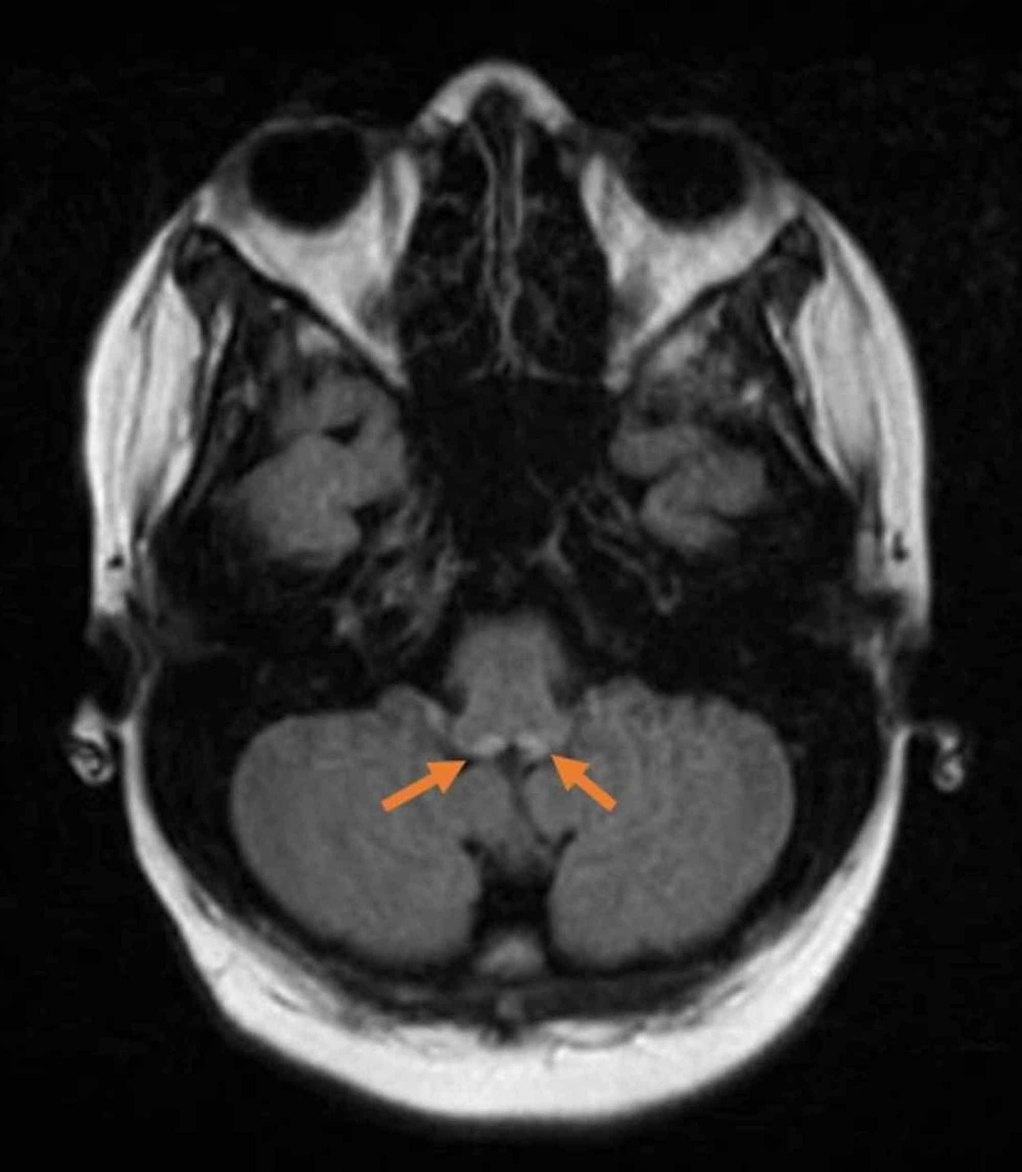
MRI brain non-contrast showing T2 signal abnormality in medial thalami bilaterally Equivocal signal abnormality in periaqueductal gray nuclei and increased signal present within medial vestibular nuclei in the dorsal medulla as seen in Wernicke's encephalopathy.

Vital signs at the time of presentation were as follows: heart rate of 160 beats/minute, blood pressure of 160/90 mm Hg, the temperature of 98.7 Fahrenheit, and respiratory rate of 18 breaths/minute. Physical examination was remarkable for disorientation to time and place, bilateral horizontal nystagmus with restricted abduction in both eyes, mild dysmetria, and broad-based ataxic gait. No abdominal distension or tenderness or rash or skin lesions was noted. Initial laboratory examination revealed white blood cell count of 16000 (reference range: 4000-11000 per microlitre), hemoglobin of 14 g/dl (normal: 12-16 g/dl), blood glucose 174 mg/dl (normal: 70-110 mg/dl), lactic acid of 6.7 mmol/l (normal: 0.5-1.6 mmol/l), albumin of 3 g/dl (reference range: 3.5-5.2 g/dl). Liver function tests were increased as well (Table 1). Acetaminophen levels were undetectable, and the toxicology screen was negative. The initial computed tomography (CT) of the head was negative for intracranial pathology. She was admitted to the intensive care unit for further workup of encephalopathy and elevated liver enzymes. A lumbar puncture (LP) was unrevealing. The electroencephalogram (EEG) did not identify any seizure activity. Magnetic resonance imaging (MRI) showed a symmetrical signal abnormality in the pulvinar and dorsomedial region of the thalami consistent with WE (Figure 1). A provisional diagnosis of WE was made based on clinical evidence of ataxia, confusion, ophthalmoplegia, and findings on imaging. The diagnosis was confirmed by identifying a low vitamin B1 level of 41.5 nmol/l (normal: 74-222nmol/l). She was started on peroral thiamine replacement 500 mg three times daily, followed by 250 mg daily for five days. This resulted in a dramatic improvement in her mentation and vision.

**Table 1:** Trend of liver function tests during hospitalization LT - alanine aminotransferase; AST - aspartate aminotransferase; ALP - alkaline phosphatase; INR - internationalized normal ratio; N/A - not available

	ALT (normal: 0-41U/L)	AST (normal: 0-41U/L)	Total bilirubin (normal: 0.2-1.2mg/dl)	ALP (normal: 30-115U/L)	INR (normal: 0.65-1.30)	Albumin (normal: 3.5-5.2g/dl)
On admission	614	220	1.7	120	1.81	4.0
Hospital day 3	268	67	3.9	66	1.19	2.4
Hospital day 8	347	130	1.6	68	0.96	2.9
Hospital day prior to discharge	51	95	0.4	90	N/A	4.7

**Figure 2:**
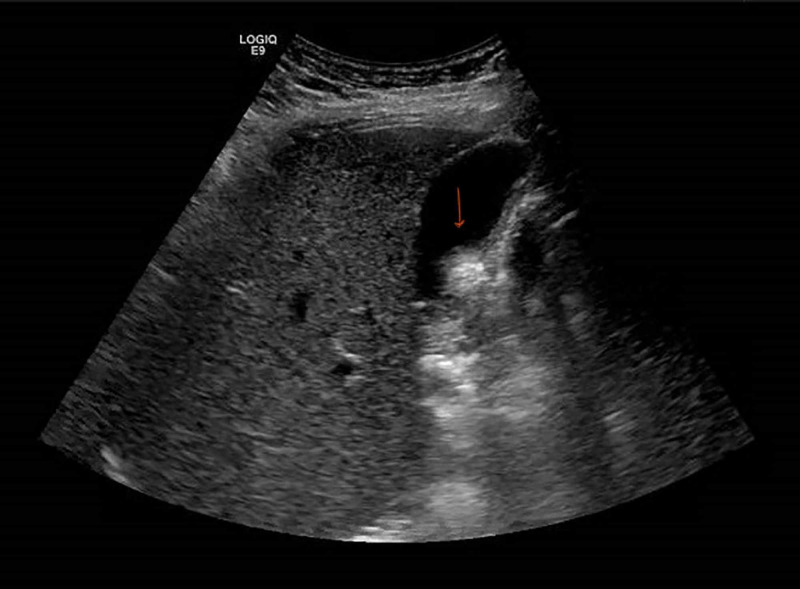
Ultrasound of right upper quadrant of abdomen showing cholelithiasis with normal liver echogenicity

Work up for transaminitis including hepatitis panel (HBsAg, HBsAb, HBcAb, immunoglobulin M/immunoglobulin G, hepatitis C antibody, hepatitis E, hepatitis A) serum ferritin, transferrin saturation, ceruloplasmin level, anti-nuclear antibody, smooth muscle antibody, gamma globulin, anti-mitochondrial antibody, herpes simplex virus, human immunodeficiency virus, cytomegalovirus, and Epstein-Bar virus serologies were negative. Ultrasound abdomen showed cholelithiasis, with normal echogenicity of the liver (Figure 2). Liver enzymes trended down gradually during the hospital stay, and she was discharged 15 days after admission on oral thiamine supplements.

## Discussion

The Wernicke Korsakoff syndrome is a known neurologic complication of thiamine deficiency [5]. It includes acute WE, requiring emergent treatment and Korsakoff syndrome, a chronic neurological condition develops because of WE. WE is mostly seen in chronic alcoholics, but can also occur in non-alcoholic patients with prevalence ranging from 0.04% to 0.13% [6]. Predisposing conditions include end-stage renal disease, chronic malnutrition, malabsorption, diarrhea, vomiting, bariatric surgery, and increased metabolic requirements caused by pregnancy [7].

Bariatric surgeries can be classified as malabsorptive, restrictive, or combined (Table 2) [8]. Weight loss occurs as a result of remodeling of gastrointestinal physiology from alterations in the structure of the gastrointestinal tract during which food is bypassed from the stomach and duodenum. Long term results of pathophysiologic alterations after these procedures are still not well understood [9]. Even though thiamine deficiency resulting in WE is most commonly reported after Roux-en-Y gastric bypass [10], it can also occur after sleeve gastrectomy despite it being a restrictive procedure with near-normal intestinal absorption. Possible explanations for this include noncompliance, inadequate diet, or prolonged vomiting as in our patient [11]. 

**Table 2:** Types of bariatric procedures

Restrictive bariatric procedures	Malabsorptive bariatric procedures	Combined bariatric procedures
Adjustable gastric banding and gastric balloons	Ileal interposition	Roux-en-Y gastric bypass
Vertical sleeve gastrectomy	Biliopancreatic diversion	Biliopancreatic diversion with duodenal switch

It is also well known that thiamine requirements are high in pregnancy due to increased demand. Increased requirements in pregnancy are thought to result from the sequestration of the vitamin by the fetus and placenta. Hyperemesis gravidarum (HG) is a complication of pregnancy characterized by severe nausea, vomiting, and ketosis, which is usually seen in 0.3 to 2.3% of pregnant women [12]. It is also associated with thiamine deficiency due to decreased oral intake from prolonged nausea and vomiting. HG, in addition to causing electrolyte abnormalities such as hyponatremia, hypokalemia, metabolic alkalosis, can also cause elevated liver enzymes, as was noted in our patient. Abnormal liver function tests have been reported in nearly 50% of patients with HG [13]. Liver function abnormalities usually return to normal levels within a few days of volume expansion and the cessation of vomiting.

Thiamine is a water-soluble vitamin. The half-life of thiamine is 10 to 20 days. The average time in which deficient patients develop symptoms is from four to 12 weeks, as shown in several case reports and systematic reviews [14]. Thiamine pyrophosphate is the biologically active form of vitamin B1; it is a cofactor for several key enzymes in many biochemical pathways in the brain, including transketolase, alpha-ketoglutarate dehydrogenase, and pyruvate dehydrogenase. The mechanism by which neurological damage occurs is unknown but is believed to be due to inhibition of thiamine promoted biochemical pathways.

The clinical presentation depends on the baseline levels of thiamine stores before surgery and post-surgery compliance with vitamin supplementation. Diagnosis of WE is often missed as patients do not always present with the classic triad of encephalopathy, oculomotor features (nystagmus, conjugate gate palsies, ophthalmoparesis) and gait ataxia. Typically only 46% of patients who underwent sleeve gastrectomy with WE had all three findings [15]. The most common presentation is altered mental status, as in our case. Atypical symptoms include optic neuropathy, papillary edema, motor, and sensory polyneuropathy, vestibular defects, asterixis, deafness were also reported [14].

Diagnostic workup includes laboratory studies such as serum thiamine levels and red blood cell (RBC) transketolase activity. Usually, the levels are low but can be normal [16]. When a diagnosis of WE is suspected, immediate thiamine replacement takes precedence over laboratory findings. Imaging studies are not necessary for all patients and shouldn’t delay treatment. However, imaging such as MRI or CT head is useful in providing additional evidence and to rule out other conditions. MRI (93% specificity and 53% sensitivity) is more sensitive than a CT scan for diagnosis of WE [17]. The most common MRI findings include hyperintense signals in the dorsal medial thalamic nuclei, a periaqueductal gray area, third and fourth ventricle, as seen in our case [18].

Administration of thiamine is a simple, inexpensive, and effective treatment. Patients with suspected WE require immediate intravenous (IV) administration of 500 mg three times daily for two consecutive days, followed by 250 mg peroral for an additional five days [19]. Thiamine should be administered before glucose to prevent the worsening of WE. Daily oral administration of 100 mg should be continued after completion of IV treatment until patients are no longer at risk.

## Conclusions

In summary, we describe a case of WE diagnosed by clinical symptoms and radiological findings in a pregnant woman four months after she underwent a sleeve gastrectomy. Even though laparoscopic sleeve gastrectomy is a restrictive bariatric procedure, nutritional deficiencies are still possible, especially in patients with other risk factors such as pregnancy, hyperemesis gravidarum, and decreased oral intake as in our case. WE from thiamine deficiency is a serious neurological complication that may lead to chronic irreversible neurological damage and even death with delayed diagnosis and treatment. Patients undergoing any bariatric procedure must have a multidisciplinary team approach, including baseline nutritional evaluation, pre- and post-operative periods, and at minimum, yearly follow up.
